# Age, but Not Experience, Affects Courtship Gene Expression in Male *Drosophila melanogaster*

**DOI:** 10.1371/journal.pone.0006150

**Published:** 2009-07-07

**Authors:** Elizabeth A. Ruedi, Kimberly A. Hughes

**Affiliations:** 1 Program in Ecology and Evolution, University of Illinois at Urbana-Champaign, Urbana, Illinois, United States of America; 2 Department of Biological Science, Florida State University, Tallahassee, Florida, United States of America; Indiana University, United States of America

## Abstract

Mutation screens in model organisms have helped identify the foundation of many fundamental organismal phenotypes. An emerging question in evolutionary and behavioral biology is the extent to which these “developmental” genes contribute to the subtle individual variation that characterizes natural populations. A related question is whether individual differences arise from static differences in gene expression that arose during previous life stages, or whether they are due to dynamic regulation of expression during the life stage under investigation. Here, we address these questions using genes that have been discovered to control the development of normal courtship behavior in male *Drosophila melanogaster*. We examined whether these genes have static or dynamic expression in the heads of adult male flies of different ages and with different levels of social experience. We found that 16 genes of the 25 genes examined were statically expressed, and 9 genes were dynamically expressed with changes related to adult age. No genes exhibited rapid dynamic expression changes due to social experience or age*experience interaction. We therefore conclude that a majority of fly “courtship” genes are statically expressed, while a minority are regulated in adults with respect to age, but not with respect to relevant social experience. These results are consistent with those from a recent microarray analysis that found none of the canonical courtship genes changed expression in male flies after brief exposure to females.

## Introduction

Mutation screens in model organisms have uncovered the building blocks of many fundamental phenotypes [reviewed in 1], [2], [3]. These experiments reveal which genes and gene interactions are necessary for the production of a wild-type phenotype. An emerging question for evolutionary and behavioral biologists is the extent to which these genes also contribute to the subtle individual variation that characterizes natural populations [e.g.], [4], [5], [6,7]. A related question is whether these individual differences arise from “static” baseline differences that arose during previous life stages, or whether they are due to “dynamic” gene regulation during the life stage under investigation.

Behavioral variation is known to arise from both statically- and dynamically-expressed genes [e.g.], [8], [9,10]. Static differences leading to behavioral variation is seen in the *foraging* gene of the fruit fly, *Drosophila melanogaster*, with two known functional alleles under balancing selection (*for^R^* and *for^S^*), which correspond to the rover and sitter foraging phenotypes in larvae and adults [11]. The *rover* allele results in consistently higher mRNA levels of the *for* gene and higher levels of cGMP-dependent protein kinase (PKG) activity than the *sitter* allele, in juvenile and adult stages [9]. The *for* gene also appears to be dynamically-regulated by food intake in *Drosophila* [12], [13]. The honeybee (*Apis mellifera*) ortholog of the *foraging* gene provides an example of dynamically-regulated gene expression as a function of age. An age-dependent increase in the expression of this gene occurs when worker bees transition from young hive-bound nurses to older foragers [8]; artificial stimulation of PKG activity in young bees accelerates the behavioral transition to foraging.

Experience-dependent regulation of expression is another potential source of individual variation. In the cichlid fish, *Haplochromis burtoni*, the expression of Gonadotropin-Releasing Hormone 1 (*GnRH1*) is socially regulated. Dynamic changes in the expression of *GnRH1* influence a switch from aggressive to submissive behaviors as a result of social cues [10]; some individuals are more likely to exhibit aggressive/dominance behaviors in response to those social cues than others.

Despite these noteworthy exceptions, little is known about the effects of age and experience on regulating genes that influence complex behaviors. Experiments that simultaneously manipulate both effects are rare [14]. To examine the combined effects of age and experience on the expression of genes that influence a complex behavior, we investigated genes known to affect male courtship in *Drosophila melanogaster*, which is one of the best-characterized behavioral patterns in a model organism [reviewed in 15], [16], [17]. While mutation screens have revealed many genes that are necessary for normal courtship, the normal age- and experience-dependent expression patterns of these genes are generally unknown. Understanding the expression pattern of these genes (courtship foundation, or CF genes) in response to age and environment is critical to investigating their contributions to natural variation in male courtship behavior [18], [19]. We hypothesized that these genes would be dynamically regulated by age and by social experience based on evidence for alterations in neural physiology with age and experience [20], [21], and for genetic variation in age-related male reproductive physiology [22], [23].

## Methods

### Experimental Organisms

Flies used in this study were derived from a wild population of *D. melanogaster* collected in Terhune, New Jersey in 1999 by Valerie Pierce (NJ population). This population originated from 8,000 offspring of 4,000 wild-caught females, and it has been maintained as a large, randomly mating population since that time [see 24], [25]. We obtained 500 flies from Dr. Allen Gibbs in 2003. Since that time, we have maintained the NJ population at a census size of approximately 12,000 individuals with overlapping generations and random mating, on a 12∶12 hour light:dark cycle at 25°C on standard cornmeal-molasses media that is replaced every 14 days.

To increase our power to detect age- and experience-related changes in gene expression, we used genetically identical but non-inbred males. We first created isogenic lines by choosing virgin females randomly from the NJ population. The X, II, and III chromosomes from these flies were extracted and made isogenic by Dr. Jenny Drnevich using balancer chromosomes and standard *Drosophila* crossing schemes [26]. The balancer stocks used in creation of these lines were FM7a and T(2;3)A1-W, In(2L)Cy, In(2R)Cy, Cy^1^ L^1^: TM2/T(2;3)Ubx^B18^, In(2LR)bw^V1^, bw^V1^ Sb^1^. We confirmed that the lines used in this study were homozygous at 7 highly variable microsatellite loci spread throughout the genome; these loci included BIB, CAD, DROGPAD, and MAM on chromosome II, and DMCATHPO, DMU1951, and DROLMALK on chromosome III [27].

To insure our results were not confounded by inbreeding effects, the experimental males were the offspring of a cross between two different isogenic lines (17 and 77). Virgin females from line 17 were crossed to males from line 77 in plastic bottles containing 25 flies of each sex. First instar larvae were collected from each of 30 different bottles on agar plates supplied with yeast paste. Two groups of 25 larvae were collected per bottle, and each group was transferred to a rearing vial supplied with standard cornmeal-molasses media. This controlled-density rearing was used to minimize individual variation arising from rearing conditions.

### Age and experience treatments

Our aim was to investigate gene expression differences due to age and experience with conspecifics. We therefore examined flies from four categories representing all combinations of two different ages and two different levels of social experience. Age/experience categories included: 3-day old, reproductively mature males [22] that had been exposed to mature females (mature and experienced, “ME”) or not (mature but naïve, “MN”); and 1–1.5 hour old, immature males that had been exposed to mature females but had not courted them (immature having encountered a female, “IE”) or that had no experience with females (immature and naïve, “IN”). In the NJ population, reproductively immature males can begin to exhibit courtship behavior as early as two hours post-eclosion (E.A.R., personal observation); however, their degree of progression through the courtship behaviors and their subsequent success with females at this age has not been determined.

To control for non-experimental environmental or circadian differences, fly collection, treatments, and dissections were timed so that all tissue samples could be collected simultaneously, within a 30-minute time period on a single day. Males for all four treatment groups were collected as virgins 0–1 hours post-eclosion from the controlled larval density vials, using light CO_2_ anesthesia. Males destined for ME and MN categories were collected two days prior to those destined for IE and IM categories so that treatments, dissections, and RNA extraction could be conducted simultaneously for all groups. Immediately after collection, males destined for the immature categories were placed into individual vials (IN males, N = 44) or into vials containing one previously-mated female from a stock of *ebony* (*e/e*) flies (IE males, N = 44). Males destined for the mature categories were placed into individual vials (MN males, N = 44), or into observation chambers (ME males, N = 40) so that we could insure that these males experienced courtship, but not mating [19]. Use of previously-mated females as the social stimulus for experienced-category males (IE and ME) reduced the likelihood that these males would achieve mating because mated females are resistant to mating with courting males [28]. Cotton-polypropylene stoppers used to top the vials were inserted so that the distance from the food media to the stopper was 2 cm; this was done to insure that males and females were in close proximity in the experienced categories. Observation chambers were designed to be as similar as possible to vials used in the other treatments, including the size of chamber, while also allowing many male-female pairs to be observed simultaneously [see ref. 19, for a detailed description]. Although for logistical reasons ME males experienced slightly different handling procedures from the other treatments, no significant age*experience differences in mRNA abundance were seen, and all contrasts comparing ME to other categories were non-significant (see Results). Consequently, the small handling differences do not seem to have evoked measurable changes in gene expression in this experiment.

We allowed ME males and IE males to interact with a female for 30 minutes. 30 minutes was chosen because alterations in mRNA levels in response to behavioral and social stimuli have been observed in this time frame [e.g. 18], [29], [30], [31]. After this period, all males from all categories were snap frozen using dry ice. This procedure occurred 2 hours after lights-on, when genes exhibiting circadian expression are relatively stable [32] and flies are active [33]. Heads were then dissected from the frozen flies in each age/experience category. Heads from four individual flies were pooled to yield sufficient total RNA for qRT-PCR analysis. The number of independent RNA pools assayed (biological replicates) was: MN = 11; ME = 9; IN = 11; IE = 11.

### Gene expression

We identified 25 CF genes using both a literature search and a query of FlyBase [34]. From FlyBase, we extracted 65 genes matching the Gene Ontology (www.geneontology.org) category of “male courtship behaviour” and daughter categories such as “courtship song”. Using the extensive literature [including 16], [35], [36], we narrowed this list to 25 genes for which expression in adult male heads has been documented. We limited this experiment to expression in the head because we wanted to narrow our focus to genes involved in nervous system function. The 25 CF genes, their known molecular functions, their role in male courtship behavior, and key citations are listed in Table 1.

**Table 1:** Description of courtship foundation (CF) genes, including putative molecular and behavioral function.

Gene name	Abbr.	FlyBaseID	Putative Function	Aspect Male Courtship	Citation(s)
*18 wheeler*	*18w*	4364	transmembrane receptor activity	Courtship latency	[36]
*amnesiac*	*amn*	86782	G-protein-coupled receptor binding, neuropeptide hormone activity	Courtship conditioning	[15], [17], [49]
*atonal*	*ato*	10433	DNA binding, transcription factor activity	Song	[50]
*CaMKII* *		4624	ATP binding, calmodulin binding, protein serine/threonine kinase activity	Courtship conditioning	[17]
*couch potato*	*cpo*	363	mRNA binding, nucleotide binding	Courtship vigor	[16]
*courtless*	*crl*	15374	ubiquitin-protein ligase activity	Courtship drive	[15], [17], [49], [51], [52]
*dunce*	*dnc*	479	cyclic-AMP phosphodiesterase activity	Courtship conditioning	[15], [17], [49]
*doublesex*	*dsx*	504	DNA binding, mRNA binding, transcription factor activity	Sex determination	[15], [17], [53], [51]
*ether a go-go*	*eag*	535	two-component sensor activity, voltage-gated potassium channel activity	Courtship conditioning	[15], [17]
*eagle*	*eg*	560	ligand-dependent nuclear receptor activity, sequence-specific DNA binding, transcription factor activity	Courtship latency, occurrence	[36]
*fruitless*	*fru*	4652	protein binding, transcription factor activity	Sex determination/Song/Sex discrimination	[53]
*homer*	*homer*	25777	receptor binding	Courtship conditioning	[35], [54]
*Kruppel homolog 1*	*Kr-h1*	28420	transcription factor activity	Possibly pheromone detection	[55]
*nonA* †		4227	mRNA binding, nucleotide binding, transcription regulator activity	Song	[15], [17], [51]
*paralytic*	*para*	3036	calcium ion binding, voltage-gated sodium channel activity	Song	[15]
*pale*	*ple*	5626	iron ion binding, tyrosine 3-monooxygenase activity	Courtship conditioning/Sex discrimination	[51]
*prospero*	*pros*	4595	transcription factor/regulator activity	Courtship drive	[56]
*quick-to-court*	*qtc*	28572	unknown	Courtship latency/Sex discrimination	[15], [51], [57]
*rutabaga*	*rut*	3301	adenylate cyclase activity, calcium- and calmodulin-responsive adenylate cyclase activity	Courtship conditioning	[15], [17], [49]
*Shaker*	*Sh*	3380	protein binding, voltage-gated potassium channel activity	Courtship conditioning	[15]
*technical knockout*	*tko*	3714	nucleic acid binding, structural constituent of ribosome	Courtship latency/Courtship Vigor	[17], [58]
*takeout*	*to*	39298	unknown	Sex determination	[46], [59]
*transformer*	*tra*	3741	transcrption factor, mRNA splicing	Sex determination/Song	[60]
*transformer2*	*tra2*	3742	mRNA binding, protein binding	Sex determination	[61]
*yellow*	*y*	4034	receptor binding, structural molecule activity, ribulose-bisphosphate carboxylase activity	Sex determination/Song/Male mating success	[51], [62], [63]

* Calcium/calmodulin-dependent protein kinase II.

† no on or off transient A.

We used quantitative real-time PCR to measure abundance of mRNA for each of the CF genes in males from each age-experience category. Forward and reverse primer sets were designed for each of the 25 CF genes using Primer Express software v2.0 (Applied Biosystems) with default “Taqman Primer and Probe Sets” settings, or using published qPCR primers [*transformer–2* and *doublesex*, 37] (Table 2). If a gene had known male-specific exons or transcripts, primers were designed to specifically amplify those. Primers were checked for specificity using NCBI BLAST searches.

**Table 2:** qRT-PCR primers.

Gene	F-primer	R-primer
*18w*	GAGGAGCCGCTAGGATCGT	ATGCTGTGGTAGATGTGCTCTGA
*amn*	TCGGTTTGGGCCAACACTT	AC TTCGTGAGCACCTTCGTTTC
*ato*	AGCTCGCAACGAACTGAGGTA	CAATGGCAGTTGGTGGTGAGT
*CaMKII*	GAACGTGTGGCTTCCGTTGT	CGCCGCGCATTAAATTTCT
*cpo*	GTTGGAAGCTCACTGTCGATACA	GCATATGAAGCGTCACTTGTTTG
*crl*	ACCATCGAGCGCATTTAGGA	AACTGTTTGTATTCCGCCATCAG
*dnc*	GATCGCATACAGGTGCTTGAGA	GGCTACCCAGCGCTTGTAAA
*dsx*	TCGAACAGGGTCGCTATGG	TCTGGAGTCGGTGGACAAATC
*eag*	GGGCGTTGCCGGATCT	TCTGCCGTCGATTGATGTTG
*eg*	CCGACCGATCGCGAAGT	TTCGAAGGAGATTTCAGCAATCA
*fru*	AGCGGTCCATGTGTCCCTACT	GATGCTTCACCCGCAAATG
*homer*	CCACCAAGAATGCCATGAAA	GGAAATGGGCGACGTGTTC
*Kr-h1*	CCACAACCCGCTGGTCTAA	GCGTGCACATCCTCATCCT
*nonA*	TGCGGATGTGCAATGAGAA	TCCATCGGATCAACCAGACA
*para*	CCAGGCTTGAAGACCATCGT	CGAACACCGACAGGGAGAA
*ple*	ACTGCCCGGGACTTCCTT	AGTTAACGTGGCGCACATACTG
*pros*	TCGACCAGGAGGACAGTGAGT	CTCCACACGCTTCTGTTGGAT
*qtc*	GTACTTGGCGCGCGTAAGA	ATCCACATGGGTGCGATTCT
*rut*	TCAACGAGATTATTGCGGACTTT	CCATATAAGTGCTACCAACGGTCTT
*Sh*	ACGCCAGGTCTGACTGATCAC	GCTTCTCGAATGACTGCTGTGT
*tko*	GGCTGTGCGCGGAGTCT	GGAAGTGGTTATTAACTATTGGCTCTTCT
*to*	TTGAAGGTGGATCGGATGGT	TGTCGGTGAAGGTTAGAGTTATGC
*tra*	GCGCCAAACACTATGCGTTA	GAGCCACGGGAATCTATGTGA
*tra2*	AGGTAAGCAAAAAGCCAATGGA	TCTGGCGCTGCAATGGA
*y*	AAACTTCAGGAGCGATATAGTTGGA	GCCAGAGCTTGGTCCTTTAGTC
*RCP1 Control*	CCTGGATTTCCCTGCTGAT	TCAATTAACTCGGAATCGGA

Total RNA was extracted using PicoPure RNA extraction kits (Arcturus), using the manufacturer's protocol including treatment with DNaseI (Qiagen). RNA was quantified and checked for purity using a spectrophotometer (Nanodrop) at 260 nm. All samples had a 260/280 nm ratio of 1.8 or greater. 200 ng RNA was reverse transcribed using a mixture of 2 µL 10× first strand Arrayscript buffer (Ambion), 1 µL 10 mM dNTP mix (Applied Biosystems), 1 µL of random decamers for primers (Ambion), 0.2 µL RNase inhibitor (Applied Biosystems), and 0.2 µL 200 U/µL Arrayscript (Ambion) in DEPC-treated water. As an exogenous control, we spiked 10 ng of plant Root Cap Protein I cRNA (RCP1, accession number NM_121758, obtained from Dr. Thomas Newman) into each reaction. Reactions were incubated at 42°C for 60 minutes, then at 95°C for 5 minutes.

For each gene, a mixture of 5 µL 2× SYBR Green Master Mix (Applied Biosystems), 1 µL 10 µM F primer, and 1 µL10 µM R primer was added to 3 µL of 6× diluted cDNA from each sample. This reaction was performed in triplicate for each sample (technical replicates). Specific transcripts from each sample were quantified using the ABI Prism HT7900 sequence detection system (Applied Biosystems). Each reaction was performed with the default PCR cycle settings for 40 cycles, and a dissociation curve was added to the final cycle to confirm the absence of primer-dimers for each gene. A five-fold log-scale dilution standard curve was generated for each gene using *D. melanogaster* genomic DNA in order to perform absolute quantification for each sample. A qRT-PCR reaction for the exogenous RCP1 control gene was performed to allow for standardization of expression of the CF genes by accounting for differences between samples in the RT reactions. A five-fold log-scale dilution standard curve was generated for RCP1 using RPC1 cRNA.

### Statistical Analyses

For each gene, standardized quantity values were calculated by dividing the mean of the quantity values for the three technical replicates for each sample by the mean of the replicate values of the RCP1 exogenous control for that sample. All standardized expression data were then log-transformed to normalize the residuals in order to meet the necessary assumptions for the statistical tests (after normalization, the residuals did meet these assumptions).

#### Multivariate Analyses

To evaluate the overall effects of age and experience, we performed a multivariate analysis of variance (MANOVA) on expression of all genes simultaneously using R statistical software v. 2.5 (R Development Core Team, 2007). The model used for the MANOVA contained the effects of age, experience, and the interaction term age*experience as well as a random error term. We used the Pillai trace statistic to approximate the Type-III F-statistic in our MANOVA, which uses eigenvalues to compare the error sums of squares to the sums of squares of the hypotheses (Johnson 1998).

To examine the ability of expression profiles to appropriately classify flies into the age/experience categories, we used a linear discriminant analysis [38], [39]. The linear discriminant analysis is as effective as alternative parametric and non-parametric versions of classification analyses [40], especially in cases where only a few canonical factors explain most of the variation [41]. In addition, it allows visualization of distances between populations on a plane [41], which many other classification methods do not. To determine the number of significant linear discriminant factors in our expression data, we used SAS PROC CANDISC (SAS Institute, 2007). The eigenvalues showed that LD1 was highly significant (F_75,28_ = 5.62; P<0.0001), while LD2 was marginally non-significant (F_48,20_ = 1.88; P = 0.062) and LD3 was not significant (F_23,11_ = 1.37; p = 0.3). Thus, any discussion of LD3 should be interpreted with caution.

We performed linear discriminant analyses using the “lda” function in R (MASS package). We then used the “predict” function to classify individual flies based on resubstitution using the linear discriminate scores for all three discriminant factors, and cross-validated the classification using the “CV” command in the “lda” function. CV performs a leave-one-out cross-validation of the ability of the LD loading scores to accurately predict the class of each individual, and is a more conservative estimate than resubstitution. Finally, we visualized the expression patterns of the age/experience categories using single-factor hierarchical clustering on normalized data with Genesis software [42].

#### Single-gene analyses

To determine the significance of effects of age, experience, and the interaction between these two factors on the expression of individual genes, we performed a fixed-effect ANOVA (SAS PROC MIXED, SAS Institute, 2007) on the normalized data. Each gene was fit to a model: y = μ+*a*+*x*+a*x+*ε*, where y was the log-transformed standardized quantity of cDNA for that a particular sample, *a* was the fixed effect of age, *x* was the fixed effect of experience, a*x was the interaction effect between age and experience, and *ε* was the random error (Type III SS). False discovery rate (FDR) was calculated using the method described in Benjamini and Hochberg [43] for age, experience, and age*experience to control for multiple hypothesis tests. If genes showed no significant change in expression between any of the age/experience categories, we considered them static. If genes displayed significant changes in expression between age groups, between males housed individually or with females, or with any age/experience interaction, we considered them dynamic.

## Results

### Multivariate Analyses

The MANOVA showed a highly significant effect of age, a non-significant effect of experience, and a marginally non-significant interaction of age*experience on gene expression differences between the age/experience categories (Table 3). The linear discriminant analysis yielded three linear discriminate factors (LD factors; scores listed in Table 4). LD1 accounted for 93.3% of the total variance, while LD2 and LD3 accounted for 4.8% and 1.9%, respectively.

**Table 3:** Multivariate analysis of variance.

Effect	Pillai(df)	Approximate F(df)	P-value
*Age*	0.99(1)	48.28 (25,9)	<0.0001
*Experience*	0.76(1)	1.17 (25,9)	0.43
Age*Experience	0.87(1)	2.46(25,9)	0.08

**Table 4:** Linear discriminant loading scores.

Gene	LD1	LD2	LD3
*18w*	1.46	3.443	−4.008
*amn*	−0.499	0.722	1.938
*ato*	0.405	5.796	−1.522
*CaMKII*	−3.28	1.468	−6.462
*cpo*	2.657	−2.307	0.025
*crl*	3.857	−6.141	−1.59
*dnc*	−2.348	3.31	−3.768
*dsx*	−0.139	3.264	−0.557
*eag*	−0.603	−0.301	5.171
*eg*	−5.366	0.431	4.743
*fru*	−1.706	−3.042	1.617
*homer*	−0.925	−9.294	−0.686
*Kr-h1*	1.038	−8.492	−5.62
*nonA*	2.13	−1.062	5.847
*para*	1.242	−4.383	1.275
*ple*	−5.849	−0.354	−4.589
*pros*	−0.433	0.164	−4.068
*qtc*	−0.813	0.198	1.227
*rut*	4.429	3.229	8.839
*Sh*	−1.627	2.164	−2.815
*tko*	2.401	−0.025	−2.184
*to*	0.279	2.893	−2.159
*tra*	−2.065	5.61	−2.944
*tra2*	4.493	−1.053	5.162
*y*	−1.458	5.393	−0.309

**Figure 1:**
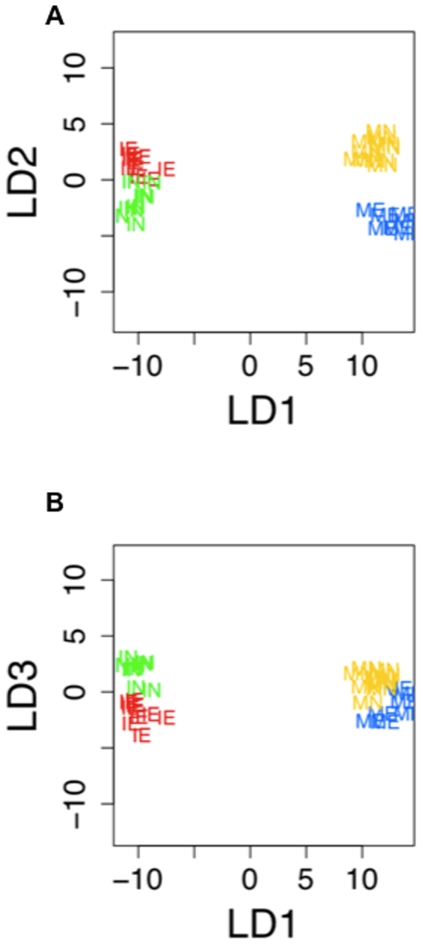
Plot of linear discriminant (LD) scores. “IN”: immature and naïve; “IE”: immature having encountered a female; “MN”: mature and naïve; “ME”: mature with courtship experience. A) LD1 vs. LD2. B) LD1 vs. LD3.

Plotting the linear discriminant scores for individual samples in each age/experience category revealed that variation in LD1 is mainly explained by differences in gene expression related to age (Figures 1A and 1B). LD2 appears to discriminate between MN+IE males and ME+IN males, reflecting the tendency of some genes to exhibit respond age-by-experience interactions (Figure 1A). Finally, LD3 discriminates between expression patterns of males who have and have not encountered females (Figure 1B). LD2 and LD3 should be interpreted cautiously, as they account for very little of the overall variance in gene expression.

Predicting classes of individuals using resubstitution of the linear discriminate scores had a high success rate (ME 100%; MN 100%; IE 100%; IN 88.9%). The more conservative cross-validation of the linear discriminate classification yielded similar results for the mature age groups (ME 71.4%; MN 70%), but was less successful for the immature age groups (IE 54.5%; IN 44.4%). The combined resubstitution and cross-validation results confirm that the linear discriminant loadings are appropriately classifying individuals based on their genes expression profiles in the majority of instances.

**Figure 2:**
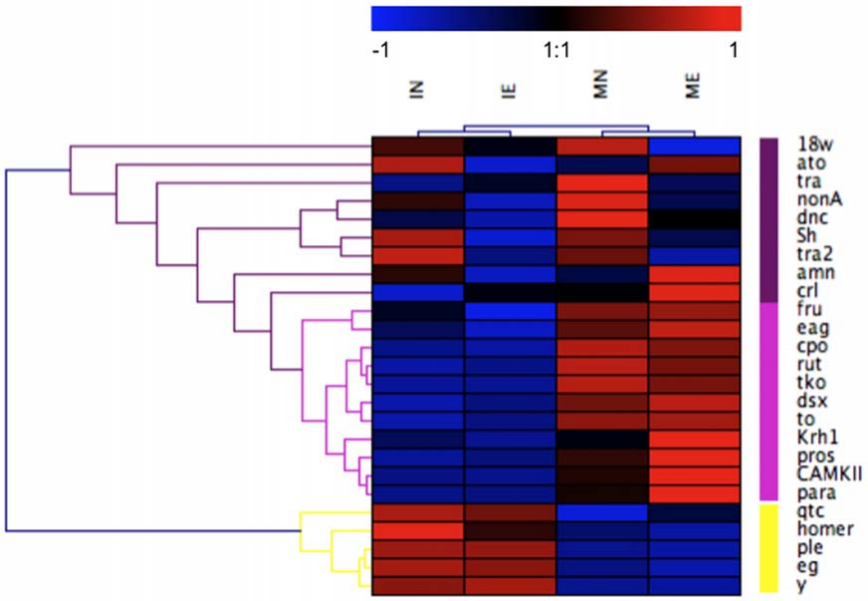
Gene expression profiles of age and experience categories. Single factor hierarchical clustering of expression profiles; red indicates increased expression, blue indicates decreased expression (as compared to the mean standardized expression taken over all genes and treatments). Three distinct clusters were found, and are identified by the color bars on the right. These clusters are discussed in the text. Full gene names are listed in Table 1.

Consistent with the results of the discriminant analysis, single factor hierarchical clustering showed a tree separating experimental groups first by age, then by experience (Figure 2). There were three major gene clusters. Genes that had higher expression in immature vs. mature males had a distinct cluster (yellow cluster in Figure 2), while genes that had higher expression in mature vs. immature males separated into two other clusters. One of these clusters (light purple cluster in Figure 2) grouped genes with differences mostly due to age, much like the immature v. mature male cluster. Genes in the third cluster (dark purple cluster in Figure 2) exhibited expression differences between categories that were not associated with just age or experience, perhaps reflecting the marginally non-significant effect of the age*experience interaction.

### Single-gene Analyses

**Figure 3:**
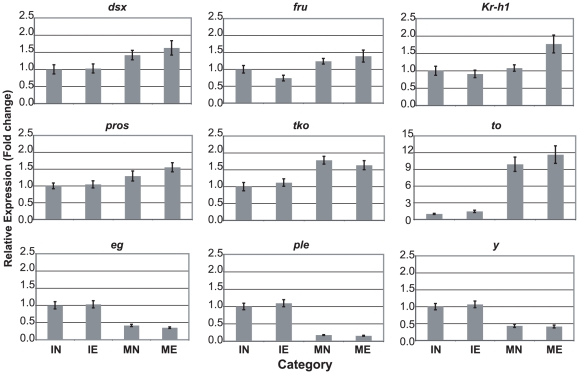
Expression levels of individual genes for each age/experience category. Relative expression levels standardized to “IN” category are reported, as well as corresponding standard errors. Only genes with significant differences in expression due to age are displayed.

Individual gene analyses revealed that, after correcting for multiple statistical tests, age had a significant effect on expression of nine of the 25 genes. There were no significant effects of experience or age*experience interaction even before correction for multiple tests (Table 5). Figure 3 displays the relative expression values and standard errors for the 9 genes with significant age effects, arbitrarily standardized to the IN category for ease of comparison Interestingly, three genes known to be part of the developmental sex determination pathway (*dsx*, *fru*, and *to*) were significantly up-regulated in older adult males.

**Table 5:** Analysis of variance on individual genes.

Gene	Age		Experience		Age*Experience	
	F-Value	Raw P-value^1^	F-Value	Raw P-value	F-Value	Raw P-value
*18w*	0.03(1,37)	0.858	0.99(1,37)	0.326	0.42(1,37)	0.523
*amn*	1.13(1,37)	0.295	0.03(1,37)	0.858	3.25(1,37)	0.08
*ato*	0.02(1,37)	0.881	0.17(1,37)	0.684	1.63(1,37)	0.21
*CaMKII*	2.49(1,37)	0.123	0.32(1,37)	0.578	0.4(1,37)	0.533
*cpo*	4.09(1,37)	0.05	0.06(1,37)	0.806	0.01(1,37)	0.923
*crl*	0.94(1,37)	0.338	0.89(1,37)	0.353	0.01(1,37)	0.924
*dnc*	0.51(1,37)	0.479	0.26(1,37)	0.616	0.05(1,37)	0.83
*dsx*	8.75(1,38)	0.005*	0.34(1,38)	0.563	0.02(1,38)	0.9
*eag*	4.7(1,37)	0.037	0(1,37)	0.985	0.63(1,37)	0.433
*eg*	47.37(1,37)	<.0001***	0.66(1,37)	0.421	0.04(1,37)	0.849
*fru*	12.36(1,36)	0.001**	1.15(1,36)	0.291	2.05(1,36)	0.161
*homer*	1.52(1,37)	0.225	0.29(1,37)	0.592	0.09(1,37)	0.772
*Kr-h1*	7.61(1,38)	0.009*	1.56(1,38)	0.219	3.5(1,38)	0.069
*nonA*	0.22(1,38)	0.641	0.77(1,38)	0.387	0.01(1,38)	0.918
*para*	4.44(1,37)	0.042	0.78(1,37)	0.384	0.7(1,37)	0.407
*ple*	234.15(1,37)	<.0001***	0.48(1,37)	0.491	0.1(1,37)	0.753
*pros*	10.65(1,37)	0.002**	1.56(1,37)	0.22	0.77(1,37)	0.387
*qtc*	2.71(1,37)	0.108	0.09(1,37)	0.772	0.38(1,37)	0.54
*rut*	4.11(1,37)	0.05	0.02(1,37)	0.901	0.11(1,37)	0.746
*Sh*	0.04(1,37)	0.839	1.75(1,37)	0.195	0.16(1,37)	0.691
*tko*	7.38(1,37)	0.01*	0.07(1,37)	0.794	0.11(1,37)	0.746
*to*	306.65(1,37)	<.0001***	3.54(1,37)	0.068	0.43(1,37)	0.515
*tra*	0.04(1,37)	0.847	0.02(1,37)	0.891	0.06(1,37)	0.805
*tra2*	0.11(1,37)	0.743	2.29(1,37)	0.139	0.01(1,37)	0.904
*y*	80.57(1,37)	<.0001***	0.03(1,37)	0.854	0.34(1,37)	0.565

1 Astericks represent significance after FDR correction for multiple hypothesis testing.

^*^p<0.05, ^**^p<0.01, ^***^p<0.001.

For logistical reasons, ME males endured slightly different handling procedures that included one additional exposure to light CO_2_ anesthesia than the other categories, as well as different environmental effects. A significant age*experience effect for a CF gene, or a significant contrast between the mean for the ME group and the other three groups might reflect expression changes due to these differences. However, no effect of age*experience was seen, and all contrasts between ME vs. MN, or ME vs. IN, IE, and MN were non-significant in the single gene analyses (P>0.05). These handling differences thus do not seem to have evoked any substantial changes in gene expression.

## Discussion

We found that courtship foundation genes are strongly affected by the age of young adult males, but affected weakly if at all by social experience. This pattern was apparent whether we analyzed genes individually or as a group. Whether or not a male had encountered or courted a female did not significantly alter gene expression, although the MANOVA and linear discriminant analysis revealed a trend for an interaction between age and experience in the expression of CF genes. Our results indicate that these genes do not exhibit dynamic expression due to the immediate effects of experience with a female or that such changes were too subtle for us to detect in this experiment. However, it is possible that some of these genes may exhibit altered expression several hours after encountering a female, or after repeated exposure to a female. *D. melanogaster* males do exhibit characteristic neural and reproductive changes during early adulthood, which might be related to these early age-dependent changes in CF genes experience based on evidence for alterations in neural physiology with age and experience [20], [21].

Carney [18] investigated whole-genome changes in gene expression of adult male *D. melanogaster* exposed to females, compared to naïve males. She exposed 4-day-old adult males to females for five minutes; any males that displayed robust courtship behavior, but did not mate, were snap frozen. Whole-body mRNA abundance of these males was compared to mRNA abundance of males not exposed to females, using whole-genome microarrays, and 43 loci changed expression consistently between the two categories. Our 25 CF genes were not among Carney's identified candidate genes responding to social behavior, further supporting our findings.

Despite several technical differences between our experimental design and Carney's, the results of the two experiments are consistent: CF genes were not found to have significant differential expression due to experience with a female. One difference between Carney's experiment and this one is that Carney investigated expression changes in whole bodies, while we concentrated on genes expressed in the head. Another difference lies is in the nature of experience with conspecifics that was manipulated. In our experiment, “inexperienced” males were housed individually, whereas Carney (2007) housed inexperienced males in groups. It has been documented that naïve males perform homosexual courtship when housed with other males [44]. Consequently males in the Carney (2007) experiment could have experienced homosexual courtship, whereas ours would not. Conversely, our “experienced” and “inexperienced” males differed with respect to exposure to females, but also differed with respect to exposure to any other adult flies, so a response to experience in our experiment need not have been due to response to an appropriate mating partner. Despite these potentially important differences, neither experiment found much evidence that CF genes respond to social experience, so these results appear to be robust, which increases our confidence in the generality of our conclusions.

In our experiment, genes that contributed most strongly to the age-related variation in expression were: *dsx*, *eg*, *fru*, *Kr*-*h1*, *ple*, *pros*, *tko*, *to*, and *y*. However, these genes did not all change in the same direction or magnitude. Three of these genes (*eg*, *ple*, *y*) were down-regulated in mature males, possibly indicating that they are important in early adult development, or that their down-regulation is necessary for normal adult function. *y* and *ple* are involved in pigmentation [reviewed in 45], which might account for their heightened expression in immature males.

The six genes that were up-regulated in mature males (*dsx*, *fru*, *Kr*-*h1*, *pros*, *tko*, *to*) might require increased expression for normal courtship or other adult behaviors. Most of these genes are transcription factors, indicating that downstream genes are also involved in male courtship. *dsx*, *fru*, and *to*, are part of the sex determination cascade, most of which is completed by eclosion [reviewed in 15], [also see 46]. However, for each of these genes, expression has been confirmed in heads of wild-type adult males [*to*: 46], [*fru*: 47], [*dsx*: 48]. Expression in adults, which we have now determined to be dynamically altered by age, supports the idea that *fru*, *dsx*, and *to* have as yet unknown functions during adulthood.

The full-genome screen performed by Carney [18] also identified experience-dependent expression changes in two genes controlled by the sex-determination hierarchy (*Obp99b* and *fit*) within five minutes of exposure to a female, further emphasizing the pleitropic role of sex-determination genes. These genes were not included in our analysis because they had not previously been identified as CF genes, but Carney's results suggest that downstream components of the sex-determination pathway respond to social experience, while our results suggest that upstream components do not.

The sixteen remaining CF genes examined in our experiment were statically expressed across age and experience level. Static expression could indicate that the CF gene is involved in development and/or maintenance of the organism and its behavior, and that it is the absence of a normal protein product during development and/or adulthood that alters male courtship. It is possible that CF genes that were statically expressed in this experiment have dynamic expression in tissue other than heads, dynamic expression in response to repeated exposure to females, or dynamic expression resulting from copulation. Therefore, we cannot extend our claim of static expression to scenarios outside these particular age/experience combinations.

Although the CF genes we examined are statically expressed in maturing males, differences in baseline expression among individual wild type flies could have important phenotypic effects. We reported recently that adult flies from a single natural population exhibit substantial genetic variation in courtship behavior under assay conditions identical to those used in this experiment [19]. If CF genes contribute to this natural behavioral variation, then the results reported here suggest that static differences in their expression or in the structure of their gene products, rather than induced differences in response to social stimuli, mediate behavioral variation. However, additional experiments will be necessary to confirm this hypothesis, because genotypes other than the one examined here could exhibit experience-mediated changes in CF gene expression. Alternatively, genes other than CF genes could mediate naturally segregating courtship variation. An experiment combining whole-genome expression analysis with behavioral assays of naturally segregating genotypes could be used to address these questions.
